# Adiponectin and Leptin—Considerations in Adult Patients with Spinal Muscular Atrophy Type 3

**DOI:** 10.3390/diagnostics15050529

**Published:** 2025-02-21

**Authors:** Marija Miletić, Zorica Stević, Stojan Perić, Milina Tančić Gajić, Jelena Rakočević, Miloš Stojanović, Bojan Marković, Miloš Žarković

**Affiliations:** 1 Clinic for Endocrinology, Diabetes and Metabolic Diseases, University Clinical Center of Serbia, 11000 Belgrade, Serbia; mtancicgajic@yahoo.com (M.T.G.); specmedico@gmail.com (M.S.); mdbojanmarkovic@gmail.com (B.M.); milos.zarkovic@gmail.com (M.Ž.); 2 Faculty of Medicine, University of Belgrade, 11000 Belgrade, Serbia; zstevic@gmail.com (Z.S.); stojanperic@gmail.com (S.P.); 3 Clinic of Neurology, University Clinical Center of Serbia, 11000 Belgrade, Serbia; 4 Institute of Histology and Embryology “Aleksandar Ð. Kostić”, Faculty of Medicine, University of Belgrade, 11000 Belgrade, Serbia

**Keywords:** spinal muscular atrophy type 3, leptin, adiponectin, adipokines

## Abstract

**Background**: Spinal muscular atrophy (SMA) is a severe neuromuscular disorder characterized by the degeneration of alpha motor neurons in the spinal cord, leading to progressive proximal muscle weakness and paralysis. SMA is clinically categorized into four phenotypes based on age of onset and motor function achieved. Patients with SMA type 3 (juvenile, Kugelberg-Welander disease) initially have the ability to walk unaided, but experience a gradual decline in motor abilities over time. However, their lifespan is not affected by the presence of the disease. Leptin, a cytokine-like hormone secreted by adipocytes, has receptors widely distributed in musculoskeletal tissues. Several studies suggest that adiponectin deficiency contributes to the development of insulin resistance, with lower adiponectin levels closely associated with greater insulin resistance and hyperinsulinemia. However, the role of adiponectin in different types of sarcopenia and its connection to insulin sensitivity remains controversial. The purpose of this study was to measure leptin and adiponectin levels in patients with SMA type 3 and explore their association with markers of insulin sensitivity. **Methods**: This cross-sectional study included 23 adult patients with SMA type 3 (SMA group) and 18 community-based healthy volunteers (control group), conducted from July 2020 to September 2024. Anthropometric parameters, body composition, body fat percentage, surrogate markers of insulin sensitivity (Homeostasis model assessment of insulin resistance index—HOMA-IR and ISI Matsuda), and circulating levels of leptin and adiponectin were measured in all participants. **Results**: Insulin resistance was present in 91.3% of patients with SMA type 3, as determined by HOMA-IR and ISI Matsuda insulin sensitivity markers. In the control group, 64.7% had insulin resistance (IR) according to HOMA-IR, while 44.4% met the ISI Matsuda criterion for IR, showing a significant difference in peripheral insulin sensitivity between groups. A significant difference in serum adiponectin levels was observed between patients with SMA type 3 and the control group, whereas there was no significant difference in serum leptin concentrations. High adiponectin levels were observed in 50% of patients with SMA type 3. In the healthy control group, adiponectin levels positively correlated with ISI Matsuda and negatively correlated with HOMA-IR, confirming the insulin-sensitizing role of adiponectin. However, this correlation was not observed in patients with SMA type 3. **Conclusions**: Our results suggest that in this specific type of hereditary neuromuscular disease, the interplay between sarcopenia and insulin leads to adiponectin resistance, challenging the canonical narrative between insulin sensitivity and adiponectin, and indicating a need for further research.

## 1. Introduction

Spinal muscular atrophy (SMA) is a severe neuromuscular disorder characterized by the degeneration of alpha motor neurons in the spinal cord, leading to progressive proximal muscle weakness and paralysis. The disease was first described in the 1890s by Werdnig [1] and Hoffmann [2]. SMA is clinically classified into four types based on the age of onset and the level of motor function achieved [3]. SMA type 3 (juvenile, Kugelberg-Welander disease) typically manifests after the age of 18 months, with patients initially able to walk independently. However, motor function tends to decline over time. Cognitive function and life expectancy are generally unaffected by the disease [4]. Dysregulated metabolism of fatty acids, impaired glucose tolerance, and mitochondrial dysfunction in muscle have already been recognized as a part of the SMA spectrum [5,6,7,8].

Given that these patients have a life expectancy comparable to the general population, we considered it essential to evaluate metabolic parameters in order to optimize their quality of life. At the same time, insights into SMA type 3 provide an opportunity to use it as an experimental model of sarcopenia, helping to uncover the complex communication between muscle and adipose tissue. As aging progresses, it is crucial to explore the various factors influencing the metabolic consequences of sarcopenia, particularly since studies on the adult population with SMA 3 are scarce.

Insulin sensitivity can be assessed by using surrogate indices such as the Matsuda index and the Homeostasis model assessment of insulin resistance index (HOMA-IR) [9,10]. The Matsuda index, which integrates both hepatic and peripheral tissue insulin sensitivity, has considerable diagnostic capacity regarding the HOMA-IR index [10,11]. In our previous study with adult patients with SMA type 3, we focused on dyslipidemia and altered glucose metabolism [12]. Studies performed by Davis et al. and Bowerman et al. have addressed the issue of insulin sensitivity among patients with different types of SMA, suggesting that the interactions involved are far more complex than the mechanical loss of muscle tissue as a target for insulin-dependent glucose uptake [6,7].

Adipose and skeletal muscle tissues form a critical metabolic link that not only supports muscle contraction but also regulates energy storage. This connection has evolved into a complex model involving metabolic substrates, adipose-derived cytokines (adipokines), and inflammatory mediators [13]. To date, more than 20 adipokines have been identified, with documented or hypothesized effects on skeletal muscle [14,15]. However, many of these adipokines show variable expression, influencing different pathways and cell types in a context-dependent manner [16].

Leptin is a cytokine-like adipokine primarily secreted by adipocytes [17]. However, previous research has shown that leptin is also produced by skeletal muscle [18,19,20,21] and bone cells [22], with abundant leptin receptors present in musculoskeletal tissues [23,24]. The leptin receptor is highly conserved across vertebrates, as leptin acts as a potent growth factor for both muscle and bone during early life stages [25]. The overall effects of leptin include appetite suppression, thermogenesis, enhanced fatty acid oxidation, and reduced body weight, primarily through its central action on the hypothalamus [26]. Adiponectin (ApN) is secreted most abundantly by white adipose tissue [27], but also by skeletal muscle cells [28], cardiomyocytes [29], parenchymal liver cells [30], and osteoblasts [31]. Adiponectin, in both its full-length and globular forms, binds to its receptors in skeletal muscles (adiponectin receptor 1, AdipoR1) and in the liver (AdipoR2) [32]. In healthy muscles, ApN regulates insulin sensitivity, inflammation, and oxidative stress. It also activates AMP-activated protein kinase (AMPK) and inhibits p70 ribosomal S6 kinase 1 (p70S6K1), enhancing insulin sensitivity. Additionally, ApN promotes the translocation of glucose transporter 4 (GLUT4) to the plasma membrane, facilitating glucose uptake [33,34]. Several studies suggest that ApN deficiency contributes to the development of insulin resistance and the pathogenesis of type 2 diabetes (DM2) [35], with lower ApN levels closely correlating with the degree of insulin resistance and hyperinsulinemia [36,37]. It has been shown that hypoadiponectinemia is associated with insensitivity to insulin, and theoretically, a high level of ApN would correlate with low HOMA-IR [38]. Insulin also reduces circulating ApN levels, suggesting that hyperinsulinemia, observed in cases of insulin resistance, could contribute to lower plasma adiponectin. However, the presence of impaired insulin post-receptor signaling in these conditions complicates the interpretation of the insulin–adiponectin relationship [26]. Some studies have reported that hyperadiponectinemia is associated with increased all-cause and cardiovascular disease (CVD) mortality [39,40,41]. Insulin resistance may play a key role in the complex relationship between serum ApN levels and aging, a phenomenon referred as “the adiponectin paradox” [42]. However, the whole spectrum of the distinctive pathways controlling adiponectin biosynthesis in vivo requires further clarification.

The aim of this study was to measure leptin and ApN levels in patients with SMA type 3 and explore their association with markers of insulin sensitivity. A review of the literature reveals that data on adipokine profile in adults with SMA type 3 are scarce, but such information could provide valuable insights into the complex interaction between adipose and muscle tissue in this type of hereditary muscle atrophy.

## 2. Materials and Methods

### 2.1. Patients’ Characteristics

This cross-sectional study included 23 patients with SMA type 3 (SMA group) and 18 community-based healthy volunteers (control group), all aged ≥18 years. The study was conducted from July 2020 to September 2024 at the Clinic of Endocrinology, Diabetes, and Metabolic Diseases, University Clinical Center of Serbia (UCCS), Belgrade, Serbia. Patients in the SMA group were followed at the Clinic of Neurology, UCCS. Genetic diagnoses were made the Center for Human Genetics at the University of Belgrade, Faculty of Biology, Belgrade, Serbia. The mean age at diagnosis was 12.8 ± 5.1 years of age. The study group consisted of 11 (47.8%) men, mean age of 41.5 ± 14.0 years, and 12 women (52.2%) with a mean age of 39.7 ± 13.1 years. Two women (16.6%) were menopausal. Patients in the SMA group were not treated with specific SMA gene therapies. At the time of endocrine investigations, 14 (60.9%) patients were wheelchair-bound, and 9 (39.1%) were using orthopedic mobility aids. The control group consisted of 18 participants, 9 men and 9 women, with a mean age of 43.7 ± 11.2 years, matched by sex, age, and BMI with SMA patients. The major exclusion criteria for all participants were known metabolic disorder or ongoing antihyperglycemic or HMG-CoA reductase inhibitors therapy. Personal medical and family history, along with anthropometric measurements (height and weight), were collected. Body mass index (BMI) was classified according to the guidelines provided by the Expert Consultative Group of the World Health Organization (WHO) [43].

Assessment of body composition and body fat percentage was performed using a Hologic Horizon^®^ dual X-ray absorption system (Hologic Inc., Bedford, MA, USA). Total body fat mass (kg), total skeletal muscle mass (kg), body fat percentage (%), and appendicular muscle mass were calculated via system software at the time of scanning. The cut-off values for body fat percentage were set at 28% for men and 40% for women [44]. The appendicular lean soft tissue index (ALSTI, kg/m^2^), calculated as the sum of lean soft tissue in the upper and lower extremities divided by height squared, was used as an approximation of muscle mass. According to the EWGSOP2 guidelines, muscle mass measured by DXA is expressed as Appendicular Skeletal Muscle Mass (ASM), with cut-off points of <20 kg for men and <15 kg for women. Alternatively, muscle mass was expressed using the Appendicular Lean Soft Tissue Index (ALSTI). The appendicular lean skeletal tissue index was calculated using the ASM/height formula with cut-off points of <7.0 kg/m^2^ for men and <5.5 kg/m^2^ for women [45].

### 2.2. Blood Analyses

Blood samples for glucose and HbA1c were collected after a 10 to 12 h overnight fast, at 8:00 am. HbA1c levels were determined using the immunoturbidimetric method on the Architect c8000 analyzer. Insulin levels were measured using the chemiluminescence immunoassay (CLIA) method on the Immulite 2000 immunoassay system (Siemens Healthcare, Erlangen, Germany).

Glucose levels ranging from 5.6 to 6.9 mmol/L or HbA1c 5.7–6.4% were considered diagnostic criteria for prediabetes. A fasting glucose concentration ≥ 7 mmol/L (126 mg/dL) or HbA1c ≥ 6.5% were diagnostic criteria for diabetes [46]. HOMA-IR was calculated using the following formula: HOMA-IR = (glucose [mmol/L] × insulin [μIU/mL])/22.5 [47].

The insulin sensitivity index-Matsuda was calculated using the following formula: ISI Matsuda index = 10,000/[√G0 × I0 × G Mean × Mean] [48]. Twelve-hour fasting serum leptin and ApN levels were measured at 8:00 a.m. using venipuncture samples, without any other dietary or activity restrictions. Plasma samples were frozen at −20 °C and analyzed using commercially available RIA Kits (Human Leptin and Human Adiponectin, Millipore, St. Charles, MO, USA). The intra- and inter-test coefficients of variation for leptin were 3.4–8.3% and 3.6–6.2%, respectively, and for adiponectin, they were 1.78–6.21% and 6.9–9.25%, respectively. The leptin/adiponectin ratio (LAR) was obtained by dividing the serum concentrations of leptin by those of adiponectin.

### 2.3. Oral Glucose Tolerance Test

The oral glucose tolerance test (OGTT) was conducted at 08:00 h following a 10 to 12 h overnight fast. After the insertion of an intravenous cannula, a baseline blood sample was collected for serum glucose and insulin measurements. Participants then consumed a 75 g oral glucose solution within five minutes, and subsequent blood samples for serum glucose and insulin were collected at 30, 60, 90, and 120 min post-ingestion. Glucose levels at 120 min between 7.8 and 11.0 mmol/L (140–199 mg/dL) and ≥11.1 mmol/L (200 mg/dL) were used as diagnostic criteria for impaired glucose tolerance (prediabetes) and diabetes, respectively [46].

### 2.4. Statistical Analysis

Spinal muscle atrophy is a rare disease in general population. Additionally, SMA type 3 is a subtype of this disease, making it a rare form as well. For this type of research, we have used convenience sampling from all patients with SMA type 3 hospitalized and treated at the Clinic of Endocrinology, Diabetes, and Metabolic Diseases, University Clinical Center of Serbia (UCCS), Belgrade, Serbia, from July 2020 to September 2024 (total of 23 patients).

Normally distributed data were expressed as mean ± standard deviation, or as median and interquartile range for data with non-normal distribution. Normality was tested using a Shapiro–Wilk test and graphically (normal QQ-plot). Categorical data were presented as numbers and percentages. Correlation between the variables was assessed by Pearson’s correlation coefficient or Spearman’s rank correlation coefficient, where appropriate. *p*-values less than 0.05 were considered significant. The IBM SPSS Statistics software for Windows, Version 23.0 (Armonk, New York, NY, USA) was used for statistical analysis.

## 3. Results

Demographic and anthropometric data of patients with SMA type 3, including fasting glycemia and HbA1c, are presented in Table 1. Eleven patients (47.8%) had normal weight, ten patients (43.5%) were classified as overweight, and two patients (8.7%) were obese. When analyzing BMI by sex, three (25%) women were overweight and none were obese, while 63.3% of men were overweight and two men were obese. A significant difference in height and weight was observed between men and women (*p* < 0.001).

Demographic and anthropometric data of 23 patients with SMA type 3 compared to 18 participants in the control group are shown in Table 2.

The percentage of body fat exceeded the reference values in all patients, regardless of sex. The average body fat percentage was 47.2 ± 4.5% in women and 40.5 ± 10.4% in men.

The mean ALSTI value in men with SMA was 3.95 (3.64–5.70) kg/m^2^, while in women it was 3.06 (2.76–3.98) kg/m^2^. There was a statistically significant sex-specific difference (*p* = 0.03). All patients met the EWGSOP 2 criterion for low muscle mass.

Table 3 shows the values of glycaemia, insulin levels in 2 h OGTT, ISI Matsuda, and HOMA-IR in patients with SMA and the control group.

In OGTT, a significant difference was observed between patients with SMA type 3 and the control group in glycemic values at 90 min (*p* = 0.030) and 120 min (*p* = 0.006). Insulin values also showed a significant difference at 90 min and 120 min of the test.

Overall, 21 (91.3%) patients in the SMA group had insulin resistance by HOMA-IR criteria. Seven patients (30.4%) were diagnosed with impaired glucose tolerance, while none had DM2. In the control group, 11 participants (64.7%) were diagnosed with IR, and one had impaired glucose tolerance, with no cases of DM2. There was no significant difference in HOMA-IR between the groups (Table 3).

A statistically significant difference was found in ISI Matsuda values between the groups (*p* = 0.008). Among patients with SMA type 3, 21 (91.3%) met the ISI Matsuda criteria for insulin resistance. In the control group, eight subjects (44.4%) met the ISI Matsuda criterion for IR.

### 3.1. Leptin Levels

There was no statistically significant difference in leptin concentrations between the SMA group and the control group (*p* = 0.099). The mean serum concentration of leptin in patients with SMA was 15.82 ± 7.02 ng/mL in females and 12.74 ± 6.74 ng/mL in males, respectively. Approximately one in five patients (*n* = 4; 22.2%) had hyperleptinemia, including three females and one male. There was no significant correlation between serum leptin concentration and BMI in either women (ρ = 0.491, *p* = 0.150) or men (ρ = −0.095, *p* = 0.823). Similarly, no significant correlation was observed between leptin concentration and ALSTI in women (ρ = 0.417, *p* = 0.265) or men (ρ = 0.543, *p* = 0.266) with SMA type 3. In the control group, the mean leptin concentration was 8.45 (4.65–19.82) ng/mL, and all subjects had leptin levels within the reference range.

### 3.2. Adiponectin Levels

Patients with SMA 3 had significantly higher levels of serum ApN compared to the control group (mean 19.76 ± 7.60 μg/mL vs. 13.59 ± 6.81 μg/mL, respectively; *p* = 0.031).

Hyperadiponectinemia was detected in 50% of patients with SMA type 3 and 16.6% of the control group.

There was no significant correlation between serum ApN values and BMI in either SMA patients (r = −0.272, *p* = 0.275) or the control group (r = −0.217, *p* = 0.499). Similarly, no significant correlation was found between serum ApN and body fat percentage in SMA patients (ρ = −0.450, *p* = 0.092). Additionally, no statistically significant correlation was observed between serum ApN values and ALSTI in patients with SMA (r = 0.483, *p* = 0.068).

The association between the levels of serum leptin and ApN with HOMA-IR and ISI Matsuda in the SMA and control group is presented in Table 4.

In patients with SMA type 3, there was no statistically significant correlation between serum leptin and HOMA-IR or leptin and ISI Matsuda.

In the control group, a statistically significant negative correlation was observed between leptin concentration and ISI Matsuda (ρ = −0.641, *p* = 0.025).

In SMA patients, there was no significant correlation between serum ApN values and HOMA-IR (r = 0.088, *p* = 0.746), or between serum ApN levels and Matsuda ISI (r = 0.190, *p* = 0.481).

However, in the control group, a strong negative correlation was found between adiponectin and HOMA-IR (r = −0.708, *p* = 0.010), while a statistically significant positive correlation was observed between adiponectin concentrations and Matsuda index (r = 0.684, *p* = 0.014).

There was no significant difference in LAR between the SMA group and the control group (median 0.75 vs. 0.64, *p* = 0.832).

## 4. Discussion

As discussed in our previous study, 91.3% of patients with SMA type 3 met the HOMA-IR criteria for insulin resistance, and 30.4% had impaired glucose tolerance, while none were diagnosed with DM2 [12]. No statistically significant difference in HOMA-IR values was observed between groups. However, there was a significant difference in ISI Matsuda values (*p* = 0.008), with 91.3% of SMA patients meeting the ISI Matsuda criteria for insulin resistance compared to 44.4% in the control group. As ISI Matsuda highlights peripheral insulin resistance, the strong difference between groups is likely due to the pronounced sarcopenia observed in SMA type 3.

The mean serum leptin concentration in patients with SMA was 15.82 ng/mL in females and 12.74 in males. Overall, 22.2% of patients had hyperleptinemia (three females, one male). No significant correlation was observed between serum leptin concentration and BMI in either sex, nor was there a statistically significant correlation between leptin concentration and ALSTI in men and women with SMA type 3. The mean leptin concentration in the control group was 8.45 ng/mL, with all subjects falling within the reference range. A study by Kolbel et al. involving 43 SMA patients (13 with SMA type 3) found hyperleptinemia in 15/35 (43%) patients, with 60% of those affected being underweight and 7% obese. Hyperleptinemia was associated with SMA type and reduced motor function [49]. Similarly, a study by Djordjevic et al. included 37 patients, 22 with SMA type 2 and 15 with type 3 SMA, with 62.2% of patients in the prepubertal period [50]. This study proved a strong positive relationship between BMI z-score and leptin levels (35). This suggests that serum leptin level is not a reliable marker of disease severity in children and adolescents with SMA types 2 and 3 [50]. However, studies on leptin concentrations in adults with SMA type 3 are lacking. In a study by Park et al., a positive correlation between leptin and lean body mass was observed in men with spinal cord injuries [51]. Additionally, Rakocevic et al. investigated serum leptin concentrations and their association with metabolic syndrome in nondiabetic patients with myotonic dystrophy type 1 (DM1) [52]. It was shown that hyperleptinemia positively correlated with insulin resistance, though its clinical significance remains unclear.

Hyperadiponectinemia was observed in 50% of patients with SMA type 3. A significant difference in serum ApN concentrations was noted between the SMA and control group (*p* = 0.031). While no significant correlation between ApN and HOMA-IR was found in patients with SMA type 3, a direct interconnection was still observed. In contrast, in healthy subjects, a strong negative correlation between serum ApN and HOMA-IR was evident (r = −0.708, *p* = 0.010). Additionally, a significant positive correlation between adiponectin concentrations and ISI Matsuda was shown (r = 0.684, *p* = 0.014), which could suggest a sustained insulin-sensitizing role of ApN in the control group. Given that 50% of patients with SMA 3 had hyperadiponectinemia and simultaneously IR, these results could be interpreted in light of possible resistance to adiponectin. The resistance to ApN could be etiologically explained by possible ablation of adiponectin receptors and/or reduced signaling of ApN receptors themselves. Intramyocellular fat depots may theoretically play a role in the development of insensitivity to insulin and ApN. In individuals with a high percentage of body fat, a decline in the expression of ApN receptor mRNA (AdipoR1/R2) leads to a reduction in ApN binding, which reduces the effector function of adiponectin. Consequently, resistance to ApN along with insulin resistance is formed in a vicious circle [53]. In 2024, De Luis et al. published the first clinical trial to evaluate the potential relationship between skeletal muscle mass (determined by bioelectrical impedance (BIA) analysis) and serum ApN values in patients with obesity and metabolic syndrome (MS). The study found that serum adiponectin levels were higher in the low skeletal muscle mass index group, with a significant inverse correlation to fat mass (FM) [54]. Additionally, a recent meta-analysis involving 557 subjects with sarcopenia indicated that patients with low muscle mass tend to have higher serum adiponectin values [55]. Similarly, Harada et al. demostrated significantly higher concentrations of ApN in men with sarcopenia and cardiovascular disease [56]. Also, in a study by Nakatsudji et al. in patients with spinobulbar muscular atrophy, serum concentrations of ApN were higher compared to a control group of the same age [57]. Elevated serum ApN levels in subjects with sarcopenia were also shown in a study by Wang et al. [58]. One potential explanation suggested to clarify the aforementioned connection is the accumulation of adipose tissue within muscle mass, which may notably influence the regulation of ApN expression [27]; a decrease in ApN receptor signaling or increase in muscle catabolism may also occur due to the coexistence of other clinical entities, with all three pathophysiological entities being applicable in patients with SMA type 3. In the sarcopenic environment, clinicians should be aware of circulus vitiosus of insensitivity to insulin and adipokines profile deterioration, with possible positive outcomes achieved through lifestyle or therapeutic interventions to minimize insulin resistance. Yamauchi et al. have shown that the concomitant dysfunction of both AdipoR1 and R2 prevents the binding and function of ApN, resulting in increased triglyceride content in the tissue, inflammation, and oxidative stress, and consequently insulin resistance [59]. Tsuchida et al. showed that the expression of AdipoR1/R2 is inversely correlated with plasma insulin levels in vivo. Moreover, the expression of AdipoR1/R2 in the skeletal muscle and adipose tissue of obese rodents is significantly reduced, which is correlated with reduced binding of ApN to skeletal muscle cell membrane fractions and decreased activation of AMPK by adiponectin [60], leading to hyperadiponectinemia. A range of clinical studies have produced conflicting results regarding the utility of ApN as a biomarker of cardiovascular risk. The Rancho Bernardo study was the first long-term study to corroborate the usefulness of adiponectin as a biomarker in coronary heart disease (CHD), demonstrating that higher ApN levels were associated with a favorable cardiovascular risk profile in both sexes from the same population [61]. However, other longitudinal studies have not demonstrated a significant association between ApN levels and the incidence of coronary artery disease [62]. Contrary to expectations, these studies found that higher circulating ApN levels were associated with an elevated risk of cardiovascular disease (CVD) and increased overall mortality [63]. Age appears to be a key factor influencing circulating ApN levels [64], and it is also positively correlated with cardiovascular disease (CVD) mortality in older adults, even in the absence of pre-existing CVD [65]. The aforementioned studies vary in terms of population characteristics, underscoring the need for further investigation; however, they clearly suggest that circulating ApN levels are influenced by a wide range of factors and may play a role in mediating both harmful and protective mechanisms. Despite its inflammatory and anti-apoptotic beneficial effects, a high circulating adiponectin level should not be interpreted as a predictor of better clinical outcomes.

Clearly, this cross-sectional study cannot establish a cause-and-effect relationship or analyze patterns over a period of time. Future longitudinal studies are essential to elucidate the roles of adipokines in sarcopenia, as these could offer significant clinical value in predicting the severity of metabolic disturbances and their progression.

## 5. Conclusions

Compared to healthy control subjects, patients with SMA type 3 had significantly different values of ISI Matsuda and serum adiponectin concentration, while no significant difference was observed in serum leptin concentration. Half of the patients with SMA type 3 had elevated serum ApN levels. The positive correlation between Adiponectin and ISI Matsuda, as well as the negative correlation with HOMA-IR in the control group, likely reflects the preserved protective role of adiponectin in terms of insulin sensitivity, which was not the case in patients with SMA type 3.

Our results suggest that sarcopenia and insulin resistance intricately lead to resistance to adiponectin. This resistance may be explained by down-regulation and/or reduced signaling of adiponectin receptors.

This study is limited by a small sample size, which may limit the universality of the findings. Further research is needed to thoroughly investigate and clarify the roles of adipokines in various forms of sarcopenia. Larger-scale studies could help us predict the severity of sarcopenia, associated metabolic consequences, and the risk of sarcopenia progression.

## Figures and Tables

**Table 1:** Demographic and anthropometric data of 23 patients with SMA type 3.

Patients’ Characteristics (*n* = 23)	Overall	Male (*n* = 11; 47.8%)	Female (*n* = 12; 52.2%)	*p*-Value
Age (years)	40.6 ± 13.2	41.5 ± 14.0	39.7 ± 13.1	0.754
Weight (kg)	72.6 ± 15.9	74.8 ± 13.3	61.4 ± 7.8	<0.001
Height (cm)	168.7 ± 8.6	173.0 (170.0–175.0)	161.5 (160.0–168.7)	<0.001
Body Fat (%)	45.8 ± 8.5	40.5 ± 10.4	47.1 ± 4.5	0.098
ALSTI (kg/m^2^)	3.76 (3.03–5.45)	3.95 (3.64–5.70)	3.06 (2.76–3.98)	0.003
Parameters of glucose metabolism				
Glucose (mmol/L)	5.02 ± 0.57	5.11 ± 0.75	4.94 ± 0.34	0.049
HbA1c (%)	5.07 ± 0.36	5.10 ± 0.22	5.04 ± 0.46	0.074

Data are expressed as numbers and frequencies (%), mean ± standard deviation or median (interquartile range); ALSTI, Appendicular Lean Skeletal Tissue Index.

**Table 2:** Demographic and anthropometric data of patients with SMA type 3 and control group.

Anthropometric Data	SMA Type 3 (*n* = 23)	Control Group (*n* = 18)	*p*-Value
Age (years)	41.6 ± 13.2	43.7 ± 11.3	0.431
Weight (kg)	72.6 ± 15.9	76.7 ± 13.2	0.381
Height (cm)	168.7 ± 8.6	171.67 ± 6.0	0.221
BMI (kg/m^2^)	25.4 ± 3.8	25.8 ± 3.9	0.793
Body Fat (%)	45.8 (38.5–50.1)	NA	
ALSTI (kg/m^2^)	3.76 (3.03–5.45)	NA	

NA, not applicable.

**Table 3:** Glycaemia and insulin in 2 h OGTT, ISI Matsuda, and HOMA-IR in patients with SMA and in the control group.

Laboratory Results	SMA Type 3 (*n* = 23)	Control Group (*n* = 18)	*p*-Value
OGTT (minutes)	Glycemia (mmol/L)	Glycemia (mmol/L)	
0’	5.20 ± 0.69	5.16 ± 0.39	0.845
30’	7.81 ± 1.72	7.51 ± 1.51	0.569
60’	8.14 ± 2.16	6.78 ± 1.97	0.050
90’	7.73 ± 2.06	5.74 ± 1.68	0.030
120’	6.86 ± 1.86	5.28 ± 1.43	0.006
OGTT (minutes)	Insulin (IU/mL)	Insulin (IU/mL)	
0’	95.0 (59.2–114.4)	78.3 (47.3–113.8)	0.340
30’	668.8 (370.4–858.1)	518.4 (445.6–768.5)	0.340
60’	706.3 (376.6–1257.0)	417.9 (316.8–661.5)	0.076
90’	863.9 (430.1–1528.9)	332.4 (252.3–521.2)	0.001
120’	705.6 (328.9–1080.2)	236.5 (184.9–455.5)	0.009
Matsuda index	2.80 ± 1.53	4.62 ± 2.28	0.008
HOMA Index	3.26 ± 1.65	2.59 ± 1.22	0.171
HbA1c (%)	5.07 ± 0.36	5.18 ± 0.29	0.328

**Table 4:** HOMA-IR, ISI Matsuda and Leptin and Adiponectin association in patients with SMA type 3 and control group.

Surrogate Markers of Insulin Sensitivity	Leptin (SMA 3)	Leptin (Control Group)	Adiponectin (SMA 3)	Adiponectin (Control Group)
HOMA-IR	ρ = 0.096, *p* = 0.724	ρ = 0.477, *p* = 0.117	r = 0.088, *p* = 0.746	**r = −0.708, *p* = 0.010**
ISI Matsuda	ρ = −0.126, *p* = 0.697	**ρ = −0.641, *p* = 0.025**	r = 0.190, *p* = 0.481	**r = 0.684, *p* = 0.014**

## Data Availability

The original contributions presented in this study are included in the article. Further inquiries can be directed to the corresponding authors.
